# CFAST: a simplified and culturally adapted stroke education tool for Chinese communities

**DOI:** 10.3389/fstro.2026.1845862

**Published:** 2026-08-21

**Authors:** Haroon Khan, Yang Wang, Jing Chen, Xiao-Bing Xie, Wei Xu, Meng Li, Dong-Ling Huang, Jiannan Xiao, Chuan-Cheng Ren

**Affiliations:** 1 Department of Neurology, The Second Affiliated Hospital, School of Medicine, The Chinese University of Hong Kong, Shenzhen & Longgang District People’s Hospital of Shenzhen, Shenzhen, Guangdong, China; 2 Department of Neurology, Zhongshan Hospital, Affiliated to Fudan University, Shanghai, China; 3 Department of Neurology, Dongfang Hospital, Affiliated to Tongji University, Shanghai, China

**Keywords:** community education, emergency medical service, FAST, health literacy, stroke

## Abstract

**Background/objectives:**

Rapid recognition of stroke symptoms in Chinese populations is poor, and improving the timely and reliable identification of patients could significantly expedite the administration of stroke therapy. This study aimed to develop and evaluate CFAST (“Zhongfeng, shuo-xiao-dong” meaning “stroke, speech, smile, move”), compare its learning efficiency, comprehension, and 1-h recall with Stroke-120, and validate its performance in a larger pragmatic cohort.

**Methods:**

Phase 1 involved a comparison of standardized community sessions using CFAST (*n* = 192) or Stroke-120 (*n* = 192). The primary outcome was 1-h recall ability post-training (0–3-point checklist). The secondary outcomes were learning efficiency (time taken to recite the mnemonic) and immediate comprehension (0–3 elements explained correctly). In Phase 2, CFAST was implemented alone among 3,688 participants.

**Results:**

In Phase 1, all CFAST-trained participants (192/192; 100%) mastered the mnemonic within 5 min, compared to 3.65% (7/192) in the Stroke-120 group after a 30-min training (*p* < 0.001). When tested for comprehension, 100% of the CFAST group accurately explained the terms “speaking,” “smiling” and “moving,” whereas only 3.65% of the Stroke-120 group correctly identified the meaning of numbers associated with stroke symptoms. Short-term retention tests showed that 100% of CFAST-trained participants could accurately recall all mnemonic elements after 1 h, compared to none in the Stroke-120 group, where only 20.31% (39/192) could recall one or two items. These results were consistently replicated in a larger CFAST cohort (n = 3,688; 100% comprehension and recall, *p* < 0.001).

**Conclusion:**

The newly developed CFAST mnemonic achieved superior short-term comprehension and recall compared with Stroke-120 and demonstrated scalability across communities. Our findings support its feasibility for public stroke education in Chinese populations.

## 1 Introduction

Stroke is currently one of the major global public health challenges, as the absolute number of stroke cases and deaths continues to rise despite a decline in age-standardized incidence and mortality rates (Feigin et al., 2022; Ma et al., 2021). The economic burden of stroke is a global public health issue, which is estimated to exceed 891 billion USD per year (Golubnitschaja et al., 2024). Within three decades (1990–2019), the global stroke incidence increased by 70%, deaths by 43%, prevalence by 85%, and disability-adjusted life years lost by 32% (Owolabi et al., 2022). In China, at least one out of five deaths is due to stroke, imposing a substantial economic burden, with total costs estimated at ¥ 952.2 billion in 2018 (Ma et al., 2024; Cheng et al., 2022). Although effective recanalization therapies, including thrombolysis and thrombectomy, offer significant clinical benefits, the global rate of thrombolysis remains very low (Wu et al., 2022). In the United States, thrombolysis rates range from 3 to 8.5% (Gao et al., 2022b), whereas in China, this rate is even lower, with only 3.8% of patients with acute cerebral infarction receiving thrombolysis (Zhou et al., 2019).

Since timely thrombolysis is directly linked to patient prognosis, increasing the rate of thrombolysis could benefit a larger number of patients (Wu et al., 2025). Various programs have been developed to improve this rate, including prehospital stroke education (Rudd et al., 2016), stroke assessment in prehospital emergency care (Bao et al., 2022), and stroke mapping (Sui et al., 2021). Extensive research has demonstrated that timely recognition is the first and most critical step in managing stroke and determining outcomes (Tekyol et al., 2023). Several stroke awareness and education tools have been developed for healthcare providers and patients, including the Face, Arm, Speech, Time (FAST) test, the Recognition of Stroke in the Emergency Room, the Los Angeles Prehospital Stroke Screen, the Melbourne Ambulance Stroke Scale, the Ontario Prehospital Stroke Screening Tool, the Medic Prehospital Assessment for Code Stroke, the Cincinnati Prehospital Stroke Scale, and Stroke-120 (Rudd et al., 2016; Zhao and Liu, 2017). These educational programs have improved public awareness about stroke, leading to a higher proportion of patients presenting to the hospital within the critical time window. Consequently, the rate of thrombolysis has started to increase, enhancing the overall effectiveness of stroke treatment.

The FAST mnemonic has proven effective in the acute phase of stroke management; however, it has primarily been adapted for the English-speaking population. To date, no stroke recognition tool has been developed specifically for the Chinese-speaking population (including those who speak Mandarin and other Chinese dialects) because of the unique linguistic features of the Chinese language compared to English. Chinese speakers are not only found in China but are also widely dispersed across Southeast Asia and around the world, including the United States, where there are nearly 5 million Chinese speakers. While some Chinese speakers are fluent in English, the majority, particularly the elderly who are at a high risk for stroke, do not speak English.

In China, the numeric mnemonic Stroke-120 was created to align with the national emergency number (1-2-0). Although this program has improved public awareness, its effectiveness in enhancing knowledge retention varies widely, particularly among older and less-educated populations. Linguistic structure likely influences recall. In Mandarin, action verbs (speak, smile, and move) naturally map onto observable stroke signs of speech disturbance, facial droop, and limb weakness, potentially improving cognitive encoding compared with numeric associations. Therefore, we developed CFAST and evaluated its effectiveness by comparing it with Stroke-120 in terms of short-term learning, immediate comprehension, and 1-h recall in a pilot community. Additionally, we validated CFAST in an expanded pragmatic cohort.

## 2 Methodology

### 2.1 Development of the Chinese FAST (CFAST) version

In English, the Face, Arm, Speech, Time (FAST) test is effective for stroke recognition but lacks semantic meaning in Mandarin. A verb-based Chinese version was created to directly link each element to observable actions:

- F (Face): Facial droop is detected when a person smiles; in Chinese, “smile” is translated as “Xiao.”
- A (Arm): Limb weakness is identified by asking the person to move the arms; “move” is translated as “Dong.”
- S (Speech): Speech disturbance is assessed by asking the person to speak; “speak” is translated as “Shuo.”

Thus, the Chinese version of FAST is “Zhongfeng—shuo-xiao-dong” (meaning “stroke— speak, smile, move”). The three monosyllabic verbs are rhythmic, intuitive, and easily pronounced by both Mandarin and dialect speakers. Table 1 shows the correspondence between the original FAST and CFAST versions.

**Table 1:** Comparison of English and Chinese FAST components.

FAST components	English Word	Meaning	CFAST version	Chinese pronunciation	Objective
F	Face	Face paralysis	Smile	Xiao	Smile to check for face paralysis
A	Arm	Limb paralysis	Move	Dong	Move limbs to check for limb paralysis
S	Speech	Speech disorder	Speak	Shuo	Speak to identify speech disorders
T	Time	Timely action to call an ambulance	—	—	—

### 2.2 Folk rhyme for the CFAST

Chinese folk rhymes are widely recognized for their effectiveness in helping people remember and understand words and concepts. To reinforce CFAST, the following rhyme was composed.

*Shuo yi shuo, xiao yi xiao, dong yi dong, chu liao wen ti shi Zhong-Feng.*

English translation: *Speak a word, smile a smile, make a move; any abnormality could indicate a stroke.*

This rhyme preserves simplicity and a rhyming structure, making it easy to memorize while clearly conveying the key message of stroke recognition.

### 2.3 Visual and graphic tools

**Figure 1:**
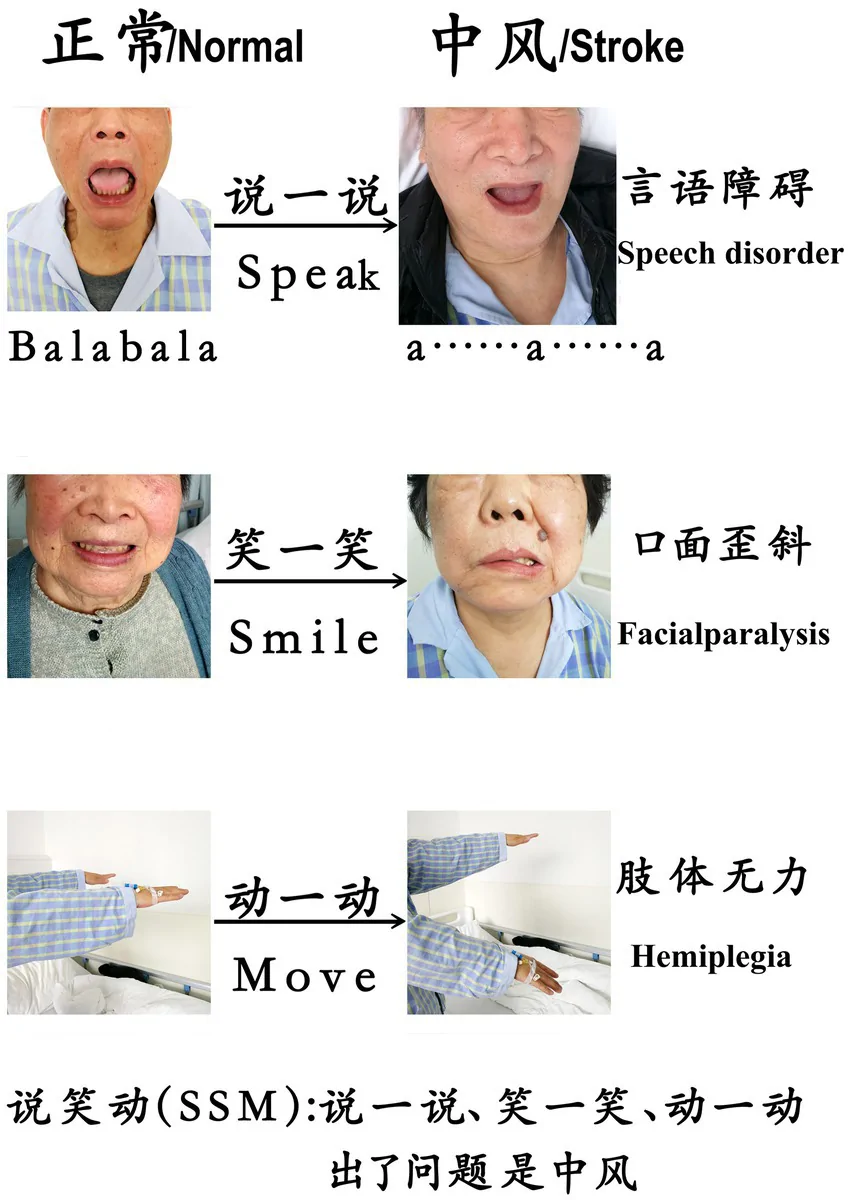
Stroke recognition using the speak-smile-move (SSM) approach. A visual guide illustrating rapid identification of stroke symptoms through three key actions—speech, facial movement, and limb motion–highlighting typical impairments associated with acute stroke.

We created a schematic diagram by capturing images of healthy elderly individuals who exhibited normal facial symmetry, clear speech, and normal limb movements. We also captured images of stroke patients exhibiting typical clinical symptoms, including facial drooping, speech impairment, and limb paralysis. All participants provided informed written consent for the use of these images. A comparative schematic diagram of these photographs is shown in Figure 1.

**Figure 2:**
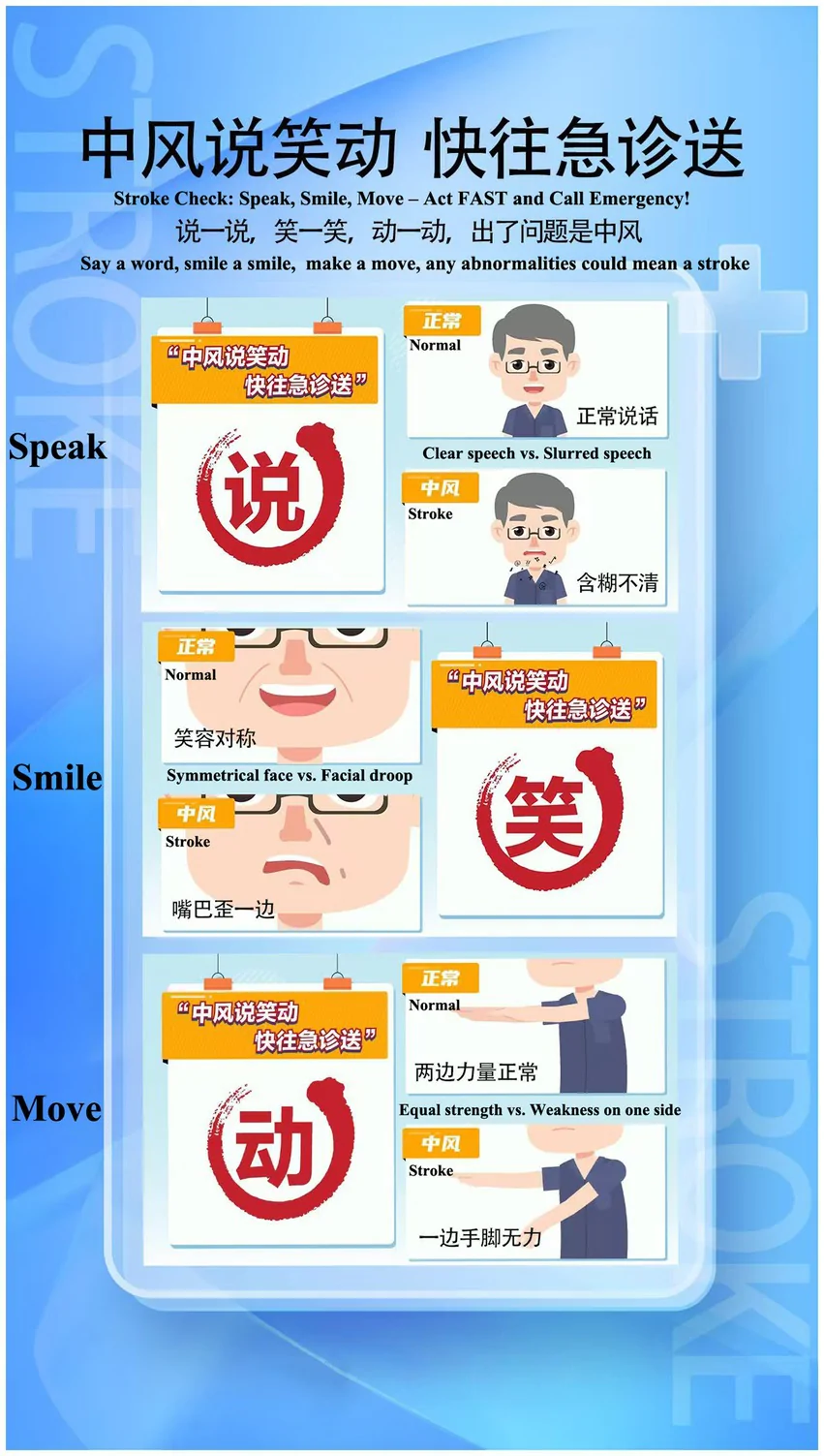
Educational infographic illustrating the speak-smile-move (SSM) method. A cartoon-based infographic developed to enhance public understanding and recognition of stroke symptoms through clear, easily interpretable depictions of speech, facial, and limb impairments.

Additionally, a cartoon-based infographic depicting the “Speak, Smile, Move” (SSM) method was designed. This visual tool has also been routinely used in hospital settings after stroke-awareness training sessions, enabling the public to quickly recognize stroke symptoms and ensure prompt emergency responses (Figure 2).

These multimodal materials reinforce comprehension during and after the training process.

### 2.4 Study design and setting

This two-phase community education study was conducted in collaboration with local healthcare service centers.

#### 2.4.1 Phase 1 (controlled comparative validation)

In total, 10 communities were non-randomly assigned to receive standardized training of either CFAST (*n* = 192) or Stroke-120 (*n* = 192) based on scheduling and feasibility.

#### 2.4.2 Phase 2 (pragmatic expansion and community education)

An additional 3,688 participants from 100 communities received CFAST-only training to educate people on CFAST and assess its scalability and reproducibility.

Phase 1 served as an initial controlled validation study comparing CFAST and Stroke-120, whereas Phase 2 functioned as a large-scale community education and validation phase focused on educating people and assessing the reproducibility and scalability of CFAST.

The primary outcome was 1-h recall of mnemonic elements, representing short-term retention of educational content. An independent facilitator assessed recall ability using a predefined 0–3 point checklist. The secondary outcomes included immediate comprehension (0–3 points based on the number of CFAST elements correctly understood and explained immediately after training) and learning efficiency, measured as the time taken for accurate unaided recitation (right-censored at 30 min).

### 2.5 Participants and recruitment

Adults (≥ 18 years) who attended community health sessions or responded via WeChat channels were invited to participate voluntarily after receiving a brief explanation of the study purpose. Participants who provided informed consent were included, while those who did not complete the study or did not provide consent for participation were excluded from the analysis. Sociodemographic information (age, sex, and education level), stroke awareness, and self-reported vascular risk factors were collected using a standardized questionnaire.

The study protocols were reviewed and approved by the Medical Ethics Committee of Dongfang Hospital, affiliated to Tongji University, Shanghai (Approval No.: (185) 2020).

### 2.6 Structured CFAST training courses

To effectively educate the participants on recognizing stroke symptoms using CFAST, the following four-step sequence was followed:

*Demonstration*: Schematic diagrams were shown comparing normal and abnormal symptoms. *Verbal introduction*: The three key words were introduced: Shuo (Speak), Xiao (Smile), and Dong (Move), linking each to a symptom.

*Rhythmic reinforcement*: The CFAST rhyme was read and recited.

*Comprehension check*: Participants repeated and demonstrated each action to verify their understanding.

Stroke-120 training sessions were conducted in accordance with the standard national training protocol (Zhao et al., 2020).

### 2.7 Interventions

#### 2.7.1 Training of educators

Before community implementation, all educators received standardized training from the study team. The training covered the fundamentals of stroke, recognition of common stroke symptoms, the principles and educational content of both CFAST and Stroke-120 programs, and the recommended emergency response to suspected stroke. Educators were also instructed on the use of the standardized PowerPoint presentation, pictorial materials, and the unified teaching script to ensure accurate and consistent delivery of educational sessions across all participating communities.

#### 2.7.2 Community education sessions

Each community session included two complementary components. The first component (approximately 10–30 min) focused on stroke symptom recognition using either the CFAST or Stroke-120 program. The second component (approximately 60 min) was about stroke risk factors, recommended lifestyle modifications, and appropriate emergency responses. All sessions were delivered by trained educators using standardized educational materials and a unified teaching script.

### 2.8 Statistical analysis

Data were analyzed using Sigma Plot (version 10.0, Systat Software, Chicago, IL, USA). Continuous data were summarized as mean ± standard deviation and compared using Student’s t-test. Categorical variables were presented as frequencies or percentages and analyzed using the chi-squared (*χ*^2^) test. A two-sided *p-*value of < 0.05 was considered statistically significant.

## 3 Results

### 3.1 Baseline characteristics of the participants

A total of 4,072 participants were included in this study. As shown in Table 2, nearly half were aged 60–70 years (48.1%), and 49.2% were men, whereas 50.8% were women. The majority of participants had a secondary or lower education level (65.5%). Regarding stroke awareness, 50.9% were familiar with the FAST mnemonic, and 63.2% knew of Stroke-120. Most participants (71.3%) correctly identified hemiplegia as a symptom of stroke, whereas a smaller proportion misattributed it to other causes. More than half (53.2%) reported knowing someone close who has experienced a stroke.

**Table 2:** Demographic and basic details of the participants.

Demographics	Frequency	Percentage
Age		
<40	693	17.0
40–60	875	21.5
60–70	1959	48.1
70–80	473	11.6
>80	72	1.8
Gender		
Men	2002	49.2
Women	2070	50.8
Educational level		
Elementary level	908	22.3
Secondary school	1758	43.2
Senior middle school	837	20.6
College education	423	10.4
Postgraduate education	146	3.6
Knowing about stroke FAST		
Yes	2071	50.9
Knowing about stroke 120		
Yes	2,574	63.2
What hemiplegia refers to		
Stroke	2,902	71.3
Lumbar diseases	404	9.9
Heart diseases	391	9.6
Brain injury	223	5.5
Others	152	3.7
Whether the participants have someone close to them who has experienced stroke		
Yes	2,167	53.2
Risk factors of stroke		
Hypertension	88	2.16
Diabetes	86	2.11
Hyperlipidemia	198	4.86
Smoking	68	1.67
Hypertension + diabetes	230	5.65
Hypertension + smoking	52	1.28
Hyperlipidemia + smoking	205	5.04
Hypertension + diabetes + hyperlipidemia	734	18.03
Hypertension + diabetes + smoking	54	1.32
Hypertension + hyperlipidemia + smoking	34	0.83
Diabetes + hyperlipidemia + smoking	186	4.57
Hypertension + diabetes + hyperlipidemia + smoking	1853	45.51
Hypertension + diabetes + hyperlipidemia + smoking + obesity	284	6.97
Whether stroke is treatable		
Yes	948	23.28
Reaction to stroke symptoms if someone close to the participant experienced symptoms suggesting a stroke		
Yes	2,439	59.9

Self-reported risk factors included hypertension, diabetes, hyperlipidemia, and smoking; 45.5% reported all four of them. Only 23.3% believed that stroke was treatable, and 59.9% indicated that they would take immediate action if someone close to them exhibited stroke symptoms.

### 3.2 Learning efficiency

We assessed learning efficiency by measuring the time required for participants to accurately recite and comprehend the stroke-recognition mnemonic. All participants trained with the CFAST version were able to correctly recite and explain the three elements (Shuo-Xiao-Dong, meaning “Speak-Smile-Move”) within 5 min. In contrast, most participants in the Stroke-120 group struggled to comprehend the numerical associations, even after a 30-min training session. This demonstrates that the CFAST approach allowed substantially faster learning, particularly among older adults, indicating greater efficiency and ease of comprehension in community education.

### 3.3 Immediate comprehension

To evaluate immediate comprehension, participants were asked to explain the meaning of each mnemonic element after training.

In the CFAST group, all participants (192/192; 100%) could accurately describe the meaning of *Shuo* (Speak), *Xiao* (Smile), and *Dong* (Move).

In contrast, only seven participants (3.65%) in the Stroke-120 group correctly explained all three numeric symbols (“1” = face droop, “2” = arm weakness, and “0” = speech disturbance). Another 49 participants (25.52%) could partially recall one or two meanings, while the majority (70.83%) were unable to establish any connection between the numbers and corresponding stroke symptoms, even after extensive instruction. These findings indicate that CFAST provided a clear, semantically intuitive learning advantage compared with the abstract numeric design of Stroke-120.

### 3.4 Short-term recall

To evaluate short-term retention, participants were tested 1 h after training, immediately following a separate lecture on stroke risk factors and prevention. The recall results showed that all participants in the CFAST group (192/192;100%) successfully recalled all three mnemonic elements and their meanings without prompting. In contrast, none of the participants in the Stroke-120 group could recall all three numeric associations. Only 39 (20.31%) remembered one or two items, while 153 (79.69%) were unable to recall any item. Detailed data are shown in Table 3. These results demonstrate the superior retention of the CFAST mnemonic, supporting its practical applicability to community education.

**Table 3:** Comparison of training effectiveness between CFAST and stroke-120.

Metric	CFAST (Phase 1, *n* = 192)	Stroke-120 (*n* = 192)	CFAST (Phase 2, *n* = 3,688)	*p*-value (Phase 1)
Learning efficiency	< 5 min	30 min	< 5 min	<0.001
Immediate comprehension	192/192 (100%) fully understood	7/192 (3.65%) fully understood. 49/192 (25.52%) partially; 136/192 (70.83%) none	3,688/3,688 (100%) fully understood	<0.001
Short-term recall	192/192 (100%) all 3 items	0/192 (0%) all 3; 39/192 (20.31%)recalled 1–2;153/192 (79.69%) none	3,688/3,688 (100%) all three items	<0.001

### 3.5 Replication and validation in the large-scale CFAST cohort (phase-2)

To evaluate reproducibility and real-world applicability, the CFAST training program was expanded to 3,688 participants across 100 additional community centers. Training in Phase 2 was conducted using the same protocol applied in the initial phase and was facilitated by certified instructors in coordination with community health-service personnel. Within 5 min of instruction, every participant was able to accurately recall and explain all elements of the mnemonic, indicating 100% learning efficiency and comprehension. One-hour post-training assessments showed complete recall of all three mnemonic elements (Shuo, Xiao, Dong), confirming short-term retention, identical to Phase 1 outcomes (*p* < 0.001 vs. baseline Stroke-120 results).

No significant differences in learning efficiency or recall were observed between communities of varying age distribution or educational level, indicating that CFAST performance was robust across demographic subgroups and independent of literacy or age-related factors. These findings support the scalability, reproducibility, and cultural adaptability of the CFAST model in large-population settings.

## 4 Discussion

Recanalization therapy, including intravenous thrombolysis and mechanical thrombectomy, has improved acute stroke management; however, its therapeutic window remains narrow, at 4.5 h for thrombolysis and 6 h for thrombectomy (Nowak et al., 2025; Prabhakaran et al., 2026). The key to increasing the success rate of recanalization is prompt hospital arrival, and the most crucial first step is recognizing the warning signs of a stroke. The present two-phase community study demonstrated that CFAST (Shuo-Xiao-Dong: Speak-Smile-Move), a linguistically intuitive and culturally adapted mnemonic, markedly improved comprehension, recall, and learning efficiency compared with the existing Stroke-120 program. All participants in the CFAST group mastered and recalled the mnemonic within 5 min, and these results were fully replicated in a large-scale community rollout, confirming the tool’s scalability and reproducibility.

There are various public health campaigns available to promote stroke awareness in English-speaking nations, including the FAST, the Recognition of Stroke in the Emergency Room, the Los Angeles Prehospital Stroke Screen, the Melbourne Ambulance Stroke Scale, the Ontario Prehospital Stroke Screening tool, the Medic Prehospital Assessment for Code Stroke, and the Cincinnati Prehospital Stroke Scale (Rudd et al., 2016). These tools have improved awareness and stroke outcomes to varying degrees across different regions.

FAST is the most widely used and internationally endorsed tool, recommended by the American Stroke Association and the American Heart Association (Powers et al., 2019). It has been successfully implemented in developed countries, such as the USA, England, Australia, and Japan (Vo et al., 2017; Dombrowski et al., 2013; Bray et al., 2011; Kawano et al., 2014). The original FAST mnemonic uses “F” for face paralysis, “A” for arm paralysis, “S” for speech disorder, and “T” for timely medical responses (Harbison et al., 2003). For the Chinese population, we linguistically adapted FAST into a culturally resonant version, replacing the English initials with intuitive action verbs: “Xiao” (Smile) for facial droop, “Dong” (Move) for limb weakness, and “Shuo” (Speak) for speech disturbance. The time element (“T”) was omitted, as the urgency of calling emergency services (120) is already well recognized. This three-syllable structure is rhythmical, easy to pronounce, and naturally integrated into everyday Chinese language, facilitating quick understanding and recall.

In China, two major stroke recognition tools are used: Stroke-120 and Stroke-112 (Zhao and Liu, 2017; Zhao et al., 2018). Stroke-120 links the numbers “1” with facial paralysis, “2” with paralysis in the arm/leg, and “0” with speech disorder. However, these numerical associations have no logical relationship between the numeral and the body part in the Chinese language, making them difficult to comprehend and remember, even for healthcare professionals. Despite the availability of training videos and online resources, Stroke-120 remains challenging for elderly and low-literacy populations. Similarly, Stroke-112 adopts a comparable numeric mnemonic, but public familiarity with this system also remains limited (Zhao et al., 2018; Melifonwu et al., 2023). In general, these numeric-based systems emphasize recall of the emergency number rather than intuitive recognition of stroke symptoms, which may reduce their effectiveness in urgent clinical situations.

BE-FAST, an alternative tool, adds “B” for balance and “E” for eyesight to the traditional FAST elements (Aroor et al., 2017). While it has gained traction in some countries, it is not widely accepted by Chinese people, especially among the elderly, many of whom are not familiar with English. Similar challenges exist in low-resource settings, such as Uganda, where healthcare providers showed limited awareness of the BE-FAST mnemonic, and caregivers were generally unfamiliar with this tool, leading to critical delays in treatment (Ssemmanda and Musubire, 2025). Indonesian data revealed that 64.6% of stroke patients arrived at the hospital beyond the 4.5-h thrombolysis window, with most delays attributed to symptom unawareness (Yang and Hartanto, 2024). Advanced solutions, such as mobile stroke units or wearable devices, are effective but have limitations, such as high cost, urban-centric deployment, and infrastructure dependencies that restrict their scalability (Krothapalli et al., 2024; Mathur et al., 2019). These results highlight the importance of linguistically simple, culturally adapted, and easily understood stroke recognition methods.

The effectiveness of CFAST likely stems from its semantic alignment with Mandarin syntax and the use of familiar action words that directly correspond to observable stroke symptoms. This verb-based structure facilitates semantic encoding, enabling participants to intuitively associate each action with a specific stroke sign, rather than relying on abstract memorization. Additionally, the integration of rhythmic folk rhymes and pictorial materials further strengthens learning by engaging both verbal and visual memory, an approach that has proven especially valuable for the elderly and those with limited literacy. These communication strategies are consistent with the use of concise, culturally familiar, and easy-to-remember messages commonly used in Chinese public health education campaigns and help explain the superior comprehension and retention observed in both phases of this study.

From a public health perspective, CFAST also demonstrated strong potential for large-scale use. In Phase II, the program was successfully implemented across 100 communities using standardized educational materials disseminated through both face-to-face sessions and the WeChat platform. High completion and retention rates suggest that CFAST can be easily incorporated into existing community health education networks. Its adaptability aligns well with the goals of the Healthy China 2030 initiative, which emphasizes accessible, community-based health promotion across the country.

CFAST proved to be simple to learn and highly memorable, enabling rapid stroke symptoms recognition. However, previous studies have shown that sociocultural and behavioral factors, including wait-and-see behavior, waiting for family members, underestimating symptom severity, a close-knit family network, and reluctance to activate emergency medical services, can contribute to prehospital delays (Dhand et al., 2019; Bouckaert et al., 2009; Gao et al., 2022a). Overcoming these barriers should be considered in future stroke education programs.

All educational sessions were delivered by trained educators using unified teaching materials and procedures to ensure consistency across communities. We did not evaluate whether the educator’s background or teaching style influenced participant engagement or learning outcomes. Future implementation studies should consider comparing different educator groups, such as healthcare professionals and community leaders, to optimize the scalability and implementation of CFAST.

Although the present study demonstrated the feasibility and effectiveness of CFAST as a community-based stroke education tool, several considerations should guide future studies. The uniformly high mastery rates may reflect the ceiling effect of the simple 0–3-point checklist. This study measured participants’ immediate comprehension and explanation of the three CFAST components, rather than merely recitation. However, we did not assess the higher-level application of this knowledge in real-world settings. Incorporating hypothetical stroke scenarios or case-based clinical vignettes to evaluate whether participants can correctly identify stroke symptoms and initiate appropriate emergency responses would be beneficial.

In addition, this study assessed only short-term retention 1 h after training and did not examine longer-duration retention or behavioral outcomes such as early hospital arrivals or increased thrombolysis rates. Finally, because most participants were recruited from urban communities, additional studies in rural or dialect-speaking populations are needed to determine the generalizability of CFAST. Future studies will extend the follow-up to determine whether repeated exposure to CFAST enhances durable awareness and improves real-world response times in acute ischemic stroke.

## 5 Conclusion

CFAST provides a linguistically natural, culturally embedded, and highly adaptable framework for stroke awareness. By transforming the English FAST mnemonic into everyday Mandarin action words, this tool bridges the gap between stroke education and public understanding. Its simplicity, high retention, and broad replicability make it well-suited to community deployment and national campaigns aimed at promoting rapid stroke recognition and timely medical intervention.

## Data Availability

The original contributions presented in the study are included in the article/supplementary material, further inquiries can be directed to the corresponding author.
